# Spontaneous pneumorrhachis, pneumomediastinum, pneumopericardium, and subcutaneous emphysema. Rare features of Hamman Syndrome

**DOI:** 10.1016/j.amsu.2022.103346

**Published:** 2022-02-05

**Authors:** Abdirahman Mohamed Hassan Dirie, Nesrin Aydın, Abdinafic Mohamud Hussein, Ahmed Adam Osman, Abdullahi Abdi Ahmed

**Affiliations:** a Resident Doctor at Pulmonology Department, Mogadishu Somali Turkey Recep Tayyip Erdoğan Training and Research Hospital, Digfer Road, Hodon, Mogadishu, Benadir, Somalia; b Pulmonology Department, Mogadishu Somali Turkey Recep Tayyip Erdoğan Training and Research Hospital, Mogadishu, Somalia; c Cardiovascular Surgery Department, Mogadishu Somali Turkey Recep Tayyip Erdoğan Training and Research Hospital, Mogadishu, Somalia; d Radiology Department, Mogadishu Somali Turkey Recep Tayyip Erdoğan Training and Research Hospital, Mogadishu, Somalia; e Thoracic Surgery Department, Mogadishu Somali Turkey Recep Tayyip Erdoğan Training and Research Hospital, Mogadishu, Somalia

**Keywords:** Spontaneous pneumorrhachis, Pneumomediastinum, Pneumopericardium, Subcutaneous emphysema, Hamman syndrome

## Abstract

**Introduction and importance:**

Pneumorrhachis (air within the spinal canal), Pneumomediastinum (abnormal air in the mediastinum), Pneumopericardium (air in the pericardial space), and Subcutaneous emphysema (air trapped under the skin) are rare conditions which are rare features of Hamman Syndrome. Some of pulmonary diseases that relate to pneumorrhachis have been reported in the literature; but Hamman Syndrome with Pneumorrhachis and Pneumopericardium due to violent coughs that triggered by tongue scraping are very rare.

**Case presentation:**

A 20-year-old male with no previous lung disease or trauma was brought to the emergency department due to acute chest pain, dyspnea, choking, syncope, and neck swelling which started after several self-induced coughs when he was brushing his tongue. Chest CT scan revealed Pneumorrhachis, pneumomediastinum, Pneumopericardium and extensive subcutaneous emphysema associated with lung contusions.

**Clinical discussion:**

Barotrauma due to violent coughs that triggered by tongue scraping may lead to lung injury resulting in Hamman Syndrome with rare features of pneumorrhachis and Pneumopericardium. To our knowledge this is the first case report of Hamman syndrome with pneumorrhachis and Pneumopericardium secondary to tongue brushing-induced lung injury in Somalia.

**Conclusion:**

Violent coughs from tongue scarping can lead to Hamman Syndrome with Pneumorrhachis and Pneumopericardium.

## Highlights

• This report is very rare.
• Is explains relation between self induced violent coughs and lung injury.
• It states rare co-existence of Hamman syndrome and Pneurrhachis with pneumopericardium.
• It emphasizes that most of pneumorrhacchis are self limited.

## 1 Introduction

Pneumorrhachis (PR) is defined as presence of air within the spinal canal (either intra- or extradural area) [1] and usually associated with traumatic spinal injuries or spinal surgery procedures. Also there is an established association between pneumorrhachis and some pulmonary diseases like asthma. PR can be caused by vaping-induced lung injury. Pneumorrhachis tends to remain localized and resolves spontaneously. Symptomatic pneumorrhachis with neurological deficits have been reported. The forceful coughing may lead to air leakage from ruptured alveoli into the mediastinal space [2]. Pneumorrhachis secondary to interstitial lung disease was reported by Sandhya et al., in 2011 [3]. Spontaneous Pneumomediastinum and Subcutaneous emphysema also known as Hamman Syndrome or (Macklin Syndrome) [4] can occur together as a labor complication and are rarely associated with Pneumorrhachis or Pneumopericardium [5]. To our knowledge there are no published case reports about pneumorrhachis secondary to forceful cough induced barotrauma triggered by tongue brushing. Here we present a previously healthy young adult male who presented with spontaneous lung injury due to forceful cough induced barotrauma and subsequently developed symptomatic Hamman Syndrome with its rare features of Pneumorrhachis and Pneumopericardium.

## 2 Case description

**Fig. 1:**
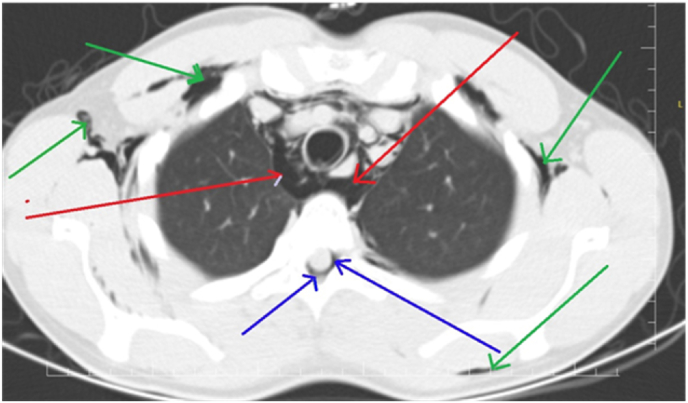
CT scan showing pneumomediastinum (red arrows), pneumorrhachis (blue arrows) and subcutaneous emphysema (green arrows). (For interpretation of the references to colour in this figure legend, the reader is referred to the Web version of this article.)

**Fig. 2:**
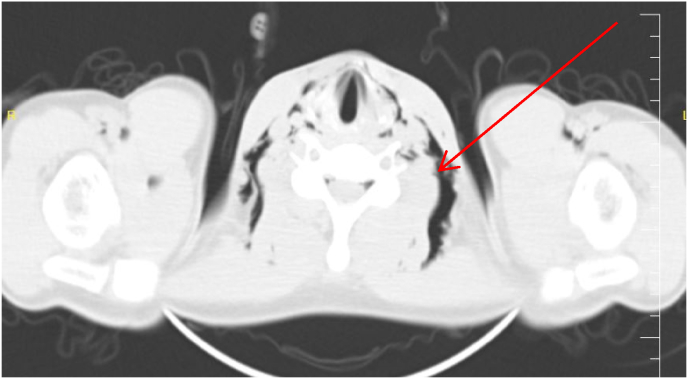
CT scan showing air collection in the bilateral neck area (arrow) indicating subcutaneous emphysema.

**Fig. 3:**
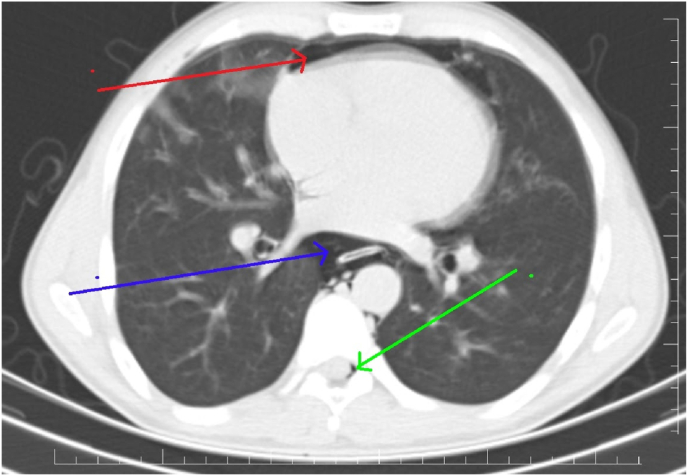
CT scan showing pneumomediastinum (blue arrow), pneumopericardium (red arrows) and pneumorrachis (green arrow). (For interpretation of the references to colour in this figure legend, the reader is referred to the Web version of this article.)

**Fig. 4:**
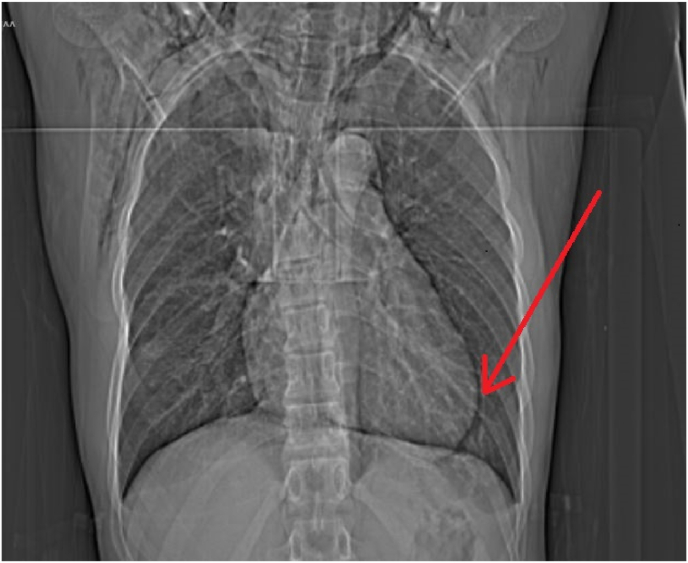
CT Scout View showing an air around the heart i.e pneumopericardium (arrow).

A 20-year-old male previously healthy, presented with chest tightness, chest pain, dyspnea associated with syncope, neck swelling, mild fever, and dysphagia for 3 hours. These symptoms started suddenly after excessive bouts of cough while he was scraping his tongue. At the time of presentation, he had mild tachycardia and tachypnea, and altered mental status with Glasgow Coma Scale of 14. In the palpation, a swelling on the base of the neck with crepitus was noted. On auscultation, there were no notable abnormalities. Neurological examination showed no focal deficit. After resuscitations and stabilization of the patient, the chest CT scan was requested which demonstrated widespread subcutaneous emphysema in the anterior chest wall, in the both axillary regions, in the both supraclavicular areas, under the skin and between the soft tissue planes. In addition to that, pneumomediastinum, pneumorrhachis, pneumopericardium, and hemorrhagic contusion in the upper lobe of the right lung were also noted but no pneumothoracic Fig. 1, Fig. 2, Fig. 3, Fig. 4. The patient was given nasal oxygen, analgesia, and intravenous fluids. Most of the symptoms decreased after 3 hours while neck swelling and mild chest pain persisted. The patient refused to be admitted to the hospital and discharged himself against medical advice. He was given oral analgesia Ibuprofen 500mg bd and PPI (pantoprazole 40mg od). After 2 weeks the patient came to the outpatient department for flow up without significant symptoms, chest x-ray and lab results did not show significant abnormalities. There was no past medical history of asthma or other lung diseases. No family history of same illness. There was no history of smoking or vaping.

## 3 Discussion

Pneumorrhachis is a potential rare condition that mostly occurs as a consequence of traumatic spinal injuries [6] or spinal surgery procedures but rarely it can occur spontaneously with pneumomediastinum and subcutaneous emphysema and this is a result of air tracking from the posterior mediastinum to the epidural or subarachnoid spaces via the neural foramina.^4^ Air typically collects in the posterior epidural space when associated with spontaneous pneumomediastinum because of its lower resistance compared with the anterior epidural space, which contains a denser vascular network. PR is usually asymptomatic and often resolves spontaneously over several days [7]. However, in rare cases, patients may present with radicular pain or neurologic deficits [8]. Pneumorrhachis can be diagnosed on a radiograph like CT scan of the spine. A CT scan is the investigation of choice in its diagnosis [9]. Management of PR is usually conservative with close monitoring for the progression of neurologic and respiratory symptoms.^8^

If there is severely symptomatic neural compression, treatment strategies include intravenous glucocorticoids, decompression by percutaneous needle aspiration, and high inspired oxygen to promote reabsorption of air [2]. Computed tomography provides reliable and prompt detection of suspected pneumorrhachis [10].

Pneumorrhachis has been shown to be associated with other pulmonary conditions, including asthma, vaping, and interstitial lung disease. The common pathophysiology is lung injury due to a high intrapulmonary pressure caused by forceful coughing leading to injury of the lung parenchyma and air leakage into the other adjacent body spaces like the spinal canal, pneumomediastinum, and subcutaneous emphysema. Chest X-ray is able to define the presence of a pneumomediastinum and subcutaneous emphysema, but the diagnosis of pneumorrhachis can only be made by CT [11]. In our opinion the hemorrhagic contusion may be caused by pressure induced capillary damage and oozing of these small vessels. The cause of altered mental status in our patient was caused by confusion but he was able to answer questions and it can be caused by hypoxia. In Somalia this is the first case of symptomatic Hamman Syndrome with Pneumorrhachis and Pneumopericardium from forceful cough induced barotrauma triggered by excessive tongue scraping. In our country the prevalence of Self-induced lung injury and its consequences are unknown. Clinical, radiological, and pathophysiological presentations of these conditions should be aware off. Obtaining a detailed history of present illness and mechanism of injury are essential in the cases presenting with symptoms of cough-induced spontaneous lung injury like dyspnea, chest tightness, and subcutaneous swellings after forceful coughing.

## 4 Conclusion

Our case suggests the risk of excessive tongue scraping with violent coughs which can lead to barotrauma and lung injury resulting in spontaneous pneumorrhachis, pneumomediastinum, pneumopericardium and subcutaneous emphysema, these are rare features of Hamman Syndrome.

## Ethical approval

In our hospital there is no ethical approval needed for case reports.

These are needed only in full articles.

## Sources of funding

There are no sponsors or any funding sources for this work.

## Author contribution

1. Abdirahman Mohamed Hassan Dirie and Nesrin Aydın; have managed the patient in the emergency roon, made follow up in the OPD, also have done a literature review, introduction and discussion writing.
2. Ahmed Adam Osman: Made a radiological diagnosis of the patient, description of the images in the manuscript.
3. Abdinafic Mohamud Hussein and Abdullahi Abdi Ahmed took part in discussion, conclusion.

## Trial registry number

Name of the registry: Not applicable
Unique Identifying number or registration ID: Not applicable
Hyperlink to your specific registration (must be publicly accessible and will be checked):

## Consent

Authors have taken written consent from the patient's mother, and it will be available on request.

## Guarantor

Abdirahman Mohamed Hassan Dirie.

Nesrin Aydin.

Ahmed Adam Osman.

Abdinafic Mohamud Hussein.

## Provenance and peer review

Not commissioned, externally peer reviewed.

## Declaration of competing interest

Authors have no any financial or personal conflict that can influence this work.
